# Advanced Therapy Medicinal Products' Translation in Europe: A Developers' Perspective

**DOI:** 10.3389/fmed.2022.757647

**Published:** 2022-02-03

**Authors:** Maja Pizevska, Jaspal Kaeda, Enrico Fritsche, Hisham Elazaly, Petra Reinke, Leila Amini

**Affiliations:** ^1^Berlin Institute of Health at Charité — Universitätsmedizin Berlin, Berlin Institute of Health Center for Regenerative Therapies, Berlin, Germany; ^2^Berlin Center for Advanced Therapies (BeCAT), Charité-Universitätsmedizin Berlin, Berlin, Germany

**Keywords:** regulatory affairs, European Medicines Agency, legislation, regulatory science, Paul-Ehrlich-Institute (PEI), advanced therapy medicinal product (ATMP), cell and gene therapies

## Abstract

Advanced Therapy Medicinal Products (ATMPs) comprising cell, gene, and tissue-engineered therapies have demonstrated enormous therapeutic benefits. However, their development is complex to be managed efficiently within currently existing regulatory frameworks. Legislation and regulation requirements for ATMPs must strike a balance between the patient safety while promoting innovations to optimize exploitation of these novel therapeutics. This paradox highlights the importance of on-going dynamic dialogue between all stakeholders and regulatory science to facilitate the development of pragmatic ATMP regulatory guidelines.

## Introduction

Navigating an Investigational Medicinal Product (IMP) through the regulatory maze to the clinic is time-consuming and expensive, with many stakeholders involved. New therapies must be rigorously tested *in-vitro* and subjected to exhaustive pre-clinical investigations in accordance with regulatory guidelines to ensure they are safe and supposedly efficacious prior to clinical trials. The existing regulatory frameworks are proving cumbersome especially when it comes to implementing first-in-human (FIH) developments and do not sufficiently reflect the great heterogeneity of the novel Advanced Therapy Medicinal Products (ATMPs). The ATMPs are logistically challenging, have complex manufacturing procedures, demanding approval processes, are highly individualized and as a consequence exceptionally expensive (1). However, ATMPs have the potential to eliminate or repair disease causing cells, offering a curative approach with opportunities to address unmet medical needs (2, 3) and the opportunity for highly personalized precision medicine. To date the majority of the currently approved ATMPs target orphan disease indications (4), but are advancing at pace such that regulatory authorities and the developers must adapt the assessment procedures and the legislation without compromising patient safety and hampering innovations (5). As such, these novel products exhibit a variety of unique characteristics that are challenging to the traditional health care systems leading to limited access by patients and, in some instances, market discontinuation (6). Global efforts are underway to improve the economic value of ATMPs by improving methods of manufacturing and adapt them for scaling (7) and advance the necessary infrastructure to treat monogenic and rare diseases (8). It is incumbent upon stakeholders to develop new tools, standards, and approaches to assess the safety, efficacy, quality, and performance of the novel pharmaceutical products (5). These tools are integral to the principles of regulatory science which ensures that data-driven policies are in place to facilitate safe and timely availability of life-saving medicines (8). Most importantly, for ATMPs to be widely available to patients worldwide, harmonizing regulatory convergence among countries should now become a priority more than ever (9), an important lesson the scientific community learned from the COVID-19 pandemic. This appraisal highlights the challenges facing regulatory science to foster science-based decision making into safeguarding public health and promoting innovation.

## The Current Regulatory Framework

In 2009 following the implementation of the Regulation 1394/2007 (10), and recognizing the innovative characteristics of ATMPs, a multidisciplinary expert committee within the European Medicines Agency (EMA), the Committee for Advanced Therapies (CAT) was established. To enable a European-wide market access, the centralized procedure on marketing authorization application (MAA) for ATMPs became mandatory, benefitting from a single evaluation process (11, 12). Additionally, in 2016 EMA launched a PRIority MEdicines (PRIME) scheme to enhance fast track development of medicines that target an unmet medical need and thereby ensure faster patient access. This accelerated pathway provides active support to efficiently develop agents for unmet medical needs and does not require large datasets. This is counterbalanced by a need for more stringent post-market safety and efficacy evaluations (13).

The clinical trial approval, evaluation and monitoring however is devolved to the individual EU member states (MS). For example, ATMP regulations in Germany are especially exacting, requiring (a) clinical trials authorization from the national competent authority (NCA) “Paul-Ehrlich Institut” (PEI); (b) approval from the local ethics committee within the state the principle investigator is located, and (c) manufacturing license authorization from the respective local competent authority (“Landesbehörde”) (14). Furthermore, the collection of starting material, e.g. peripheral blood, is subject to the German Transfusion Act (Transfusionsgesetz; TFG) (15) and/or German Transplantation legislation (16), while the local authority must approve the tissue collection site (“Entnahmeinrichtung”). If the product is considered a genetically modified organism (GMO), the PEI is responsible for environmental risk assessment in consultation with the Federal Office for Consumer Protection and Food Safety (“Bundesamt für Verbrauchschutz und Lebensmittelsicherheit”) (17).

Consequently, delays due to the variations in GMO regulation across MS result in a less-competitive and less-attractive environment for stakeholders to realize multicenter clinical trials with investigational gene therapies in Europe and has been issued by multiple stakeholders (18). Together with national competent authorities, they demand to exempt ATMPs containing or consisting of GMOs from the GMO legislation, as it has been temporarily adopted by the EU for IMPs treating or preventing COVID-19 in human (19). This exemption for IMPs to treat or prevent COVID-19 had timely and administrative benefits for the sponsors and trial sites. Stakeholders and advocates of ATMPs expect a rapid implementation of a GMO exemption scheme under the pretext of the new Clinical Trial Regulation (EU) No 536/2014 (20), which will come into force January 2022.

## Non-Clinical Regulatory Requirements for ATMPs

Given the heterogeneity and the complexity of ATMPs, which frequently involve viable cells (“living drug”), the conventional strategies designed for robust non-clinical (NC) assessment of proof-of-concept (PoC), mechanism of action (MoA), toxicology and bio-distribution are not always transferable to ATMP development. Standard non-clinical murine, *ex-vivo* assessment of dose-related safety and efficacy to test ATMPs have limited value specifically, due to the differing immunologic background and microenvironments. Elsallab et al. noted ATMPs have the disadvantage of significant uncertainties with NC translation data which may influence their benefit risk assessment. This is due to several factors, including lack of relevant animal models and clear primary pharmacological targets. Therefore, a major challenge is to identify platforms enabling rigorous evaluation of NC outcomes, which are meaningful and predictive for human clinical trials (21). To overcome these hurdles developers need to foster collaborations with industry partners and engage with regulatory agencies to define, evaluate and develop appropriate NC models where relevant data is unavailable.

## Manufacturing ATMPs

**Figure 1:**
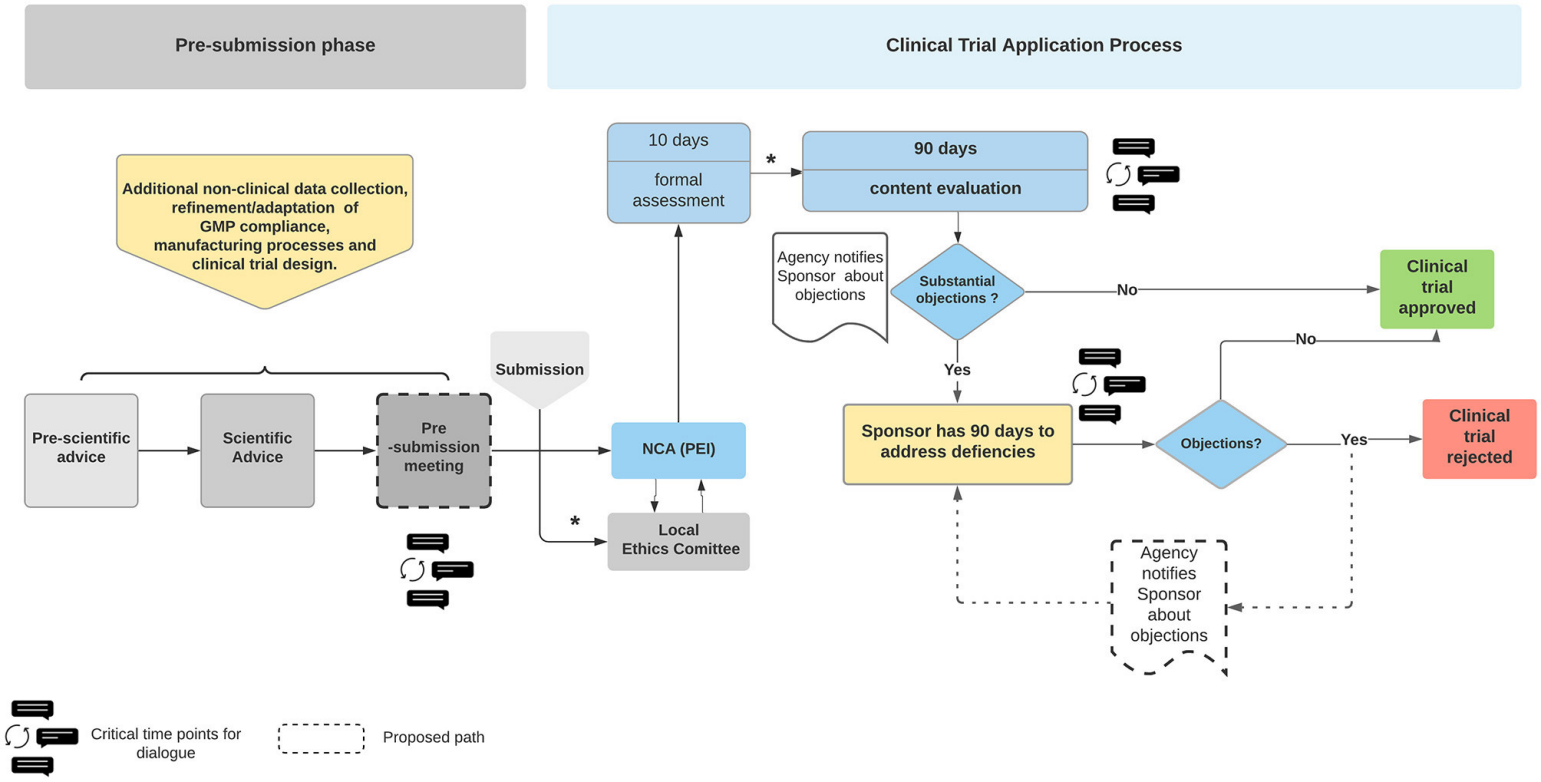
Overview of the Clinical Trial Application process for ATMPs in Germany. The Sponsor has opportunity to seek advice from the National Competent Authority (NCA), Paul Ehrlich Institute (PEI). The Sponsor is notified of the regulators' concerns at the first review discussions following formal submission. The Sponsor has 90 days to address the issues raised. The clinical trial is rejected if the regulatory body is not satisfied with the responses. The dialogue bubbles indicate the proposed timepoints for a dialogue between the regulators and sponsors. The process describes the current legislative process. The timelines may differ once the Clinical Trial Regulation EU No 536/2014 is enacted in January 2022. The boxes with dashed lines are Authors' suggestions in how to create a more dynamic dialogue, optimizing the outcome in favor of safer therapies which are available more rapidly. Blue boxes, PEI related actions; Yellow boxes, sponsor related actions. *Communication between Ethics Committee and Sponsor and intermediate steps regarding formal assessment during the CTA process are not depicted (not relevant in this context). NCA, national competent authority.

It is mandatory that ATMP manufacturing complies with good manufacturing practice (GMP) guidelines, which includes using GMP grade starting materials. But frequently, GMP grade starting material is scant and expensive. The lack of standardized regulatory framework tailored to small-scale production and for establishing specific pharmacopeia monographs for pharmaceutical grade raw materials and raw materials of biologic/human sources leads to fragmented manufacturing and impacts on quality, precision, purity, functionality, reproducibility, and stability. These challenges are often compounded by the lack of adequate expertise, technical equipment and trained personnel specific to ATMP GMP compliance. Furthermore, ATMPs are often designed for a small specific group of patients or are highly individualized. As a consequence their manufacturing is not easily amenable to GMP compliance nor automation to enable commercialization at viable cost-effective levels (22). Moreover, GMP as well as quality control guidance specific to ATMPs often lack precise details or are not suitable. For example, the guidelines EMA/CAT/80183/2014 (23) and EMA/CAT/GTWP/671639/2008 (24) include phrases such as “unless otherwise justified” or “case-by-case basis,” which leaves both the NCAs and the developers in disparity in how to interpret the legislation and implement GMP compliant strategies. Consequently, the regulatory agencies, NCA and the developers face a quandary in how to achieve balance between flexibility while aiming to provide clarity. We suggest this impasse could be circumvented by intensive interactive discussions throughout e.g., the early development process (before FIH application) involving experts during the CTA review process (see Figure 1).

As the GMP governance is entrusted to individual EU member states, the NCAs may request additional information, thus introducing another variance in applying ATMP pharmaceutical quality control across borders. In Germany, with the federal structure, the local competent authority governs GMP and grants manufacturing licenses in accordance with section 13 of the Medicinal Products Act (Ger. AMG) by the respective authority (16 in Germany) of the Federal State (Ger. *Länderbehörde*), where the manufacturing site is located (25).

To address these concerns the EudraLex Volume 4 Part IV advanced the framework for GMP-compliant ATMP manufacturing (26). While providing invaluable information and flexibility, to be applied to different cases/products of the ATMP repertoire, the built-in flexibility means the guidelines are open to interpretation and misunderstanding, which may lead to the failure to achieve the required quality standards.

## Quality Control

Quality control of ATMPs is especially complex, as they require sophisticated testing in comparison to chemical compounds, for example genetically modified cell products which are expected to bring additional potential risks to patients. In this regard, the specificity and safety of genetic modification need to be carefully examined to eliminate the risk of malignant transformation and off-target effects. These concerns were highlighted by a gene therapy trial to treat children with X-linked severe combined immunodeficiency. Some of the patients developed acute lymphoblastic T-cell leukemia following gene therapy, due to vector-mediated insertional mutagenesis (27, 28). The governance of novel technologies such as designer nucleases, e.g., CRISPR/Cas9 technology, require the development of advanced strategies to identify potential complications such as off-target editing or immunogenicity. It is challenging to definitively assess off-target and long-term effects when manipulating genes or administering genetically modified cells which can differentiate and evolve in response to surrounding stimuli (29). To their credit, the EMA's Committee for Advanced Therapies (CAT) acknowledges the need to develop regulatory guidelines covering quality, safety, and efficacy that are relevant to ATMP. Therefore, CAT proposes guidelines and opens them up for public consultation. Equally, it is being acknowledged that while *ex-vivo* gene-editing strategies may be similar, *in-vivo* gene editing requires new regulatory rules for quality, safety and efficacy testing (29). In order to realize such changes, discussions at national and EU level between the developers and regulatory authorities are needed.

## Benefit-Risk Assessment Challenges

Unlike the traditional drugs, the novel ATMPs are frequently not or cannot be tested in healthy human volunteers as is the case in the classical phase I study. ATMP trials usually are FIH studies combined as phase I/IIa and directly enroll critically ill patients. Furthermore, often only a small number of patients are included in clinical trials. Equally it is generally accepted that potential toxicities cannot be adequately addressed for ATMPs (30). Indeed, given the nature of ATMPs, benefit-risk assessment is not easily defined or measured because side effects depend on a variety of factors that are difficult to model in NC experiments. Equally, potential toxicities are rarely if at all detectable in the NC studies that are performed (31). Moreover, the availability of safety data in both, the non-clinical and clinical part is limited. Therefore the commonly recommended appropriate risk mitigation measures are especially important in ATMP clinical trials. These measures include, exceptionally close monitoring, rapidly accessible treatment options with intensive care units in close vicinity and fully trained medical professionals must be available at the trial site. Where data from animal models are available, they must be evaluated and safety data extrapolated to a FIH trial if necessary. Therefore, as mentioned above, the exceptional circumstances of ATMP development require close and regular communications in early stages of review processes between the regulatory agencies and the developers. These discussions are critical in tailoring the clinical trial accordingly (Figure 1). Experience and the relevant data gleaned from such exchanges can be chelated to formulate fit-for-purpose regulations. In this context, and as a result of a constantly changing knowledge base with time, the so called “adaptive governance” is in discussion (32), even more important since the COVID-19 pandemic. However, any novel fast access tools/mechanisms might raise concerns about the integrity of the data. As demonstrated by the public's anxiety about the speed with which SARS-CoV2 vaccines were authorized. These concerns are being addressed by longer follow-up periods and implementation of extensive post-marketing authorization studies (post authorization safety and efficacy studies). Such an approach also addresses the uncertainties about products' benefit-risk balance at the time of marketing authorization (13). Indeed, the post market authorization and approach to assess safety and efficacy in lieu of traditional randomized clinical trials is being formally explored by FDA (33, 34), commonly referred to as Real World Evidence (RWE) by analyzing Real World Data (RWD), see below.

## Regulatory Science

Regulatory science encompasses basic and applied biomedical as well as social sciences, and contributes to the development of regulatory standards and tools (35). Stakeholders within regulatory science recognize the inherent challenges in drug development and aim to bridge the gaps in the technical, regulatory, reimbursement and health technology assessment (HTA) knowledge. Such an approach is expected to enable formulation of regulations that lead to science based decision-making processes and thereby improve ATMP development and efficacy (36).

In consultation with the stakeholders, EMA refined their strategy and included a number of recommendations in the “Regulatory Science to 2025 strategy” (37). This comprises (a) establishing a multi-stakeholder forum to foster innovation in clinical trials; (b) re-enforcing relevance of patients for evidence generation; (c) promoting the use of real-world data and big data in decision-making processes; (d) providing a feasible legislative framework as well; (e) contributing to a better HTAs' preparedness and decision-making at national levels to foster innovative medicines development while enhancing translational dialogue with payers to enhance accessibility.

More recently, the STARS (**S**trengthening **T**raining of **A**cademia in **R**egulatory Science) consortium comprising 18 European regulatory agencies including EMA was established to strengthen the bidirectional dialogue between research scientists and regulatory bodies. STARS seeks to address the challenges listed above in ATMP development by first taking an inventory of the current support structures for regulatory scientific advice in academic institutions and gathering feedback on their needs. The goal is to develop a common strategy for scientific advice to be implemented by the relevant national authorities (38, 39). By establishing initiatives like STARS it provides a forum for discourse to adapt and evolve new practices and insights with the expectations that are workable and effective guidelines can be standardized across EU MS and simultaneously evolve training practices for researchers in regulatory science.

Because academia is often at the forefront in developing novel ATMP therapeutics, their input is critical in any regulatory science discussions. The academia research institutions can assist in driving the agenda *via* translational hubs as is the case in UK exemplified by “Advanced Therapy Treatment Centres” (40) and in some of the EU MS [e.g., (41–43)], but which are relatively scant in Germany. Hence, the German Research Foundation (DFG) is seeking to create an environment in which inter-medical university infrastructures across Germany can be established. Translational hubs provide an opportunity platform platform for cross fertilization of public and private institutions to advance ATMP therapeutics and make regulatory science based recommendations to committees such as CAT. In addition to academic hubs, there are examples of independent centers of excellence for example “CATAPULT—Cell and gene therapy” in the UK, which work at the interface between commercial enterprises and academia (44).

The advantages of translational hubs is exemplified by the RESTORE and ReSHAPE consortia (45, 46), which led to the development and translation of ground-breaking cellular therapies, including T cells, to modulate the immune systems in living donor transplant recipients enabling reduction of dependency upon toxic immunosuppressive drugs (47, 48). However, this also required development of new GMP compliant procedures through frequent discussions in how current regulatory guidelines should be applied and adapted where necessary.

There is a need for bi-directional discussions among all stakeholders especially during pre- and post-submission of a clinical trial application (CTA), especially when considering FIH studies (Figure 1). These exchanges would provide the applicant with an opportunity to clarify any ambiguities and identify solutions to unforeseen difficulties. Indeed, the USA Food and Drug Administration's (FDA) (49) guidance document provides a forum for the applicant to engage with the regulatory agencies to ask questions about the specific requirements and clarify any misunderstandings. These FDA-Developer discussions take place prior to submitting a final response during the CTA process, thus diminishing the possibility of approval for the trial being denied. An analysis conducted by the Alliance for Regenerative Medicine (ARM) regarding clinical trials for ATMPs in Europe supports the aforementioned concept by showing that a scientific advice prior CTA increases the speed of CT approval and decreases the questions raised by the regulatory bodies. According to this survey, the most important criteria for selecting a clinical trial site and country are the expertise of the health care professionals, quality of review and the expertise of regulatory authorities (50).

## Discussion

Heterogeneous ATMPs continue to evolve at a rapid pace, providing options for unmet clinical needs. However, the traditional approach to conducting clinical trials is not directly applicable to ATMPs requiring a change in culture. The ATMP-specific legislation is ambiguous in terms of exact requirements as highlighted above.

**Figure 2:**
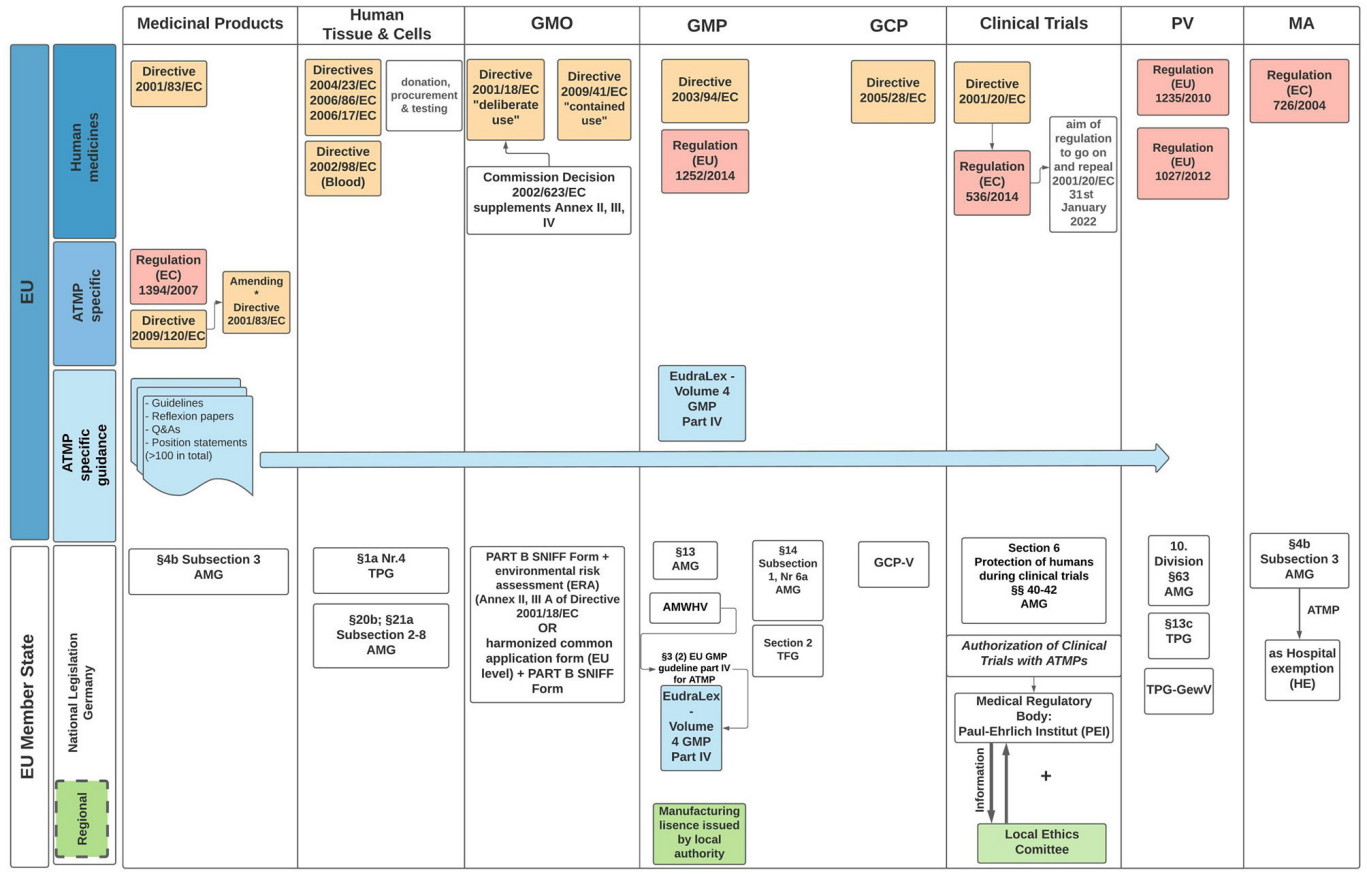
EU and German regulatory framework for ATMP specific legislation. The diagram outlines the directives EU Member States (MS) must enact, with particular reference to the regulatory guidelines applicable when seeking IMP authorization in Germany. Governance and oversight of clinical trials is the responsibility of the MS undertaking the trial. In Germany, regional authorities of the federal states are responsible for issuing a manufacturing approval. Clinical Trial authorization takes place with agreement of the local Ethics Committee. AMG, arzneimittelgesetz (*Engl*. German drug law); TPG, transplantationsgesetz (*Engl*. transplantation law); TPG-GewV, TPG—Gewebeverordnung (*Engl*. tissue regulation); TFG, transfusionsgesetz (*Engl*. Transfusion law); AMWHV, arzneimittel- und wirkstoffherstellungsverordnung (*Engl*. ordinance for the manufacture of medicinal products and active pharmaceutical ingredients); GMO, genetically modified organism (includes medicinal products with GMOs); GMP, good manufacturing practice; GCP, good clinical practice; PV, pharmacovogilance; MA, marketing authorization; Red boxes, regulations; Yellow boxes, directives; Green boxes, regional; Blue boxes, regulatory framework applicable to all EU MS.

ATMP legislation poses a dilemma in trying to balance innovative therapies requiring flexibility and provide detailed, well-defined legislation. Regulatory agencies and investigators acknowledge, ATMP oversight is obdurate as several regulatory frameworks must be considered in parallel when developing these products (Figure 2). Formulation of acceptable regulations within the EU is further complicated by the additional layer of national legislation (51). Stakeholders through translational hubs need to coalesce to define new standards with the aim of developing fit-for-purpose ATMP regulatory guidelines. Integral to this process is regulatory science as a competency within academia that could advice, formulate, and scrutinize innovative ATMP therapies. Failure to address harmonization concerns within Europe will lead to loss of expertise and innovation to USA, UK, China, and Asia more widely.

However, in the rapid changing field of ATMP stakeholders need to be supported by government finance and governance. A proactive approach by the authorities led to the development and approval of SARS-CoV2 vaccine at an unprecedented speed, without compromising patient safety. Shifting scientific advice meetings to take place online would facilitate the availability of appropriate worldwide expertise during approval discussions between stakeholders and thereby overcome ATMP development associated complexities.

Limited availability of clinical data means risk/benefit assessment is challenging. This could be addressed by gathering RWD, i.e., gathering data from numerous sources, e.g., electronic health records, medical data bases and patient information in post authorization studies. Such information provides RWE for ATMP clinical trials where the traditional randomized controlled large scale trials are not feasible or not applicable, e.g., patient population (52–57). The RWD from anti-CD19 CAR T cell therapy have demonstrated that in post marketing stages patients are much more advanced in disease, heterogeneous and the manufacturing period is longer than in the tightly controlled clinical trial setting (58, 59). The EU Commission also proposes to revise the current pharmaceutical legislative to include “new methods of evidence generation and assessment” (60).

This perspective has sought to highlight the considerable challenges stakeholders face in balancing the therapeutic potential of novel treatments while maintaining regulatory standards which are evidence based and designed to ensure patient safety. This distinction is not always absolute, requiring continuous exchange of available options with independent scientific expert advisors assisting to eliminate any ambiguity/discrepancies. Because innovations in therapeutics will continue to challenge the guidelines. The legislation must co-develop with the ATMP evolution in order to ensure the translation of innovative therapies. Support of regulatory science in the scientific field and close interaction with legislative bodies will create an environment which is more specifically tailored to the rapidly evolving needs of ATMP development to ensure more efficient market penetration for the benefit of patients.

## General Recommendations

- Encouraging effective networking between academia, industry, patient initiatives and other stakeholders.
- Emphasize and implement regulatory science as a specialized discipline.
- Support translational hubs, consortia and other structures to facilitate in bringing ATMPs to the clinic.
- Early engagement and bidirectional dialogue with the regulatory authorities.
- Enhance international harmonization efforts on ATMP legislation between EU MS and beyond.
- Recognize translation of ATMPs as a collaboration effort between all stakeholders (scientists, physicians, industry, patients AND regulatory agencies).

## Data Availability Statement

The original contributions presented in the study are included in the article/supplementary material, further inquiries can be directed to the corresponding author/s.

## Author Contributions

MP, JK, EF, HE, and LA undertook literature research and reviewed and wrote the manuscript. MP designed the Figures. PR provided the funding and reviewed the final draft. All authors contributed to the article and approved the submitted version.

## Funding

The project was partially funded from the European Union's Horizon 2020 research and innovation program under grant agreement No. 820292 (Restore) and No. 825392 (Reshape).

## Conflict of Interest

The authors declare that the research was conducted in the absence of any commercial or financial relationships that could be construed as a potential conflict of interest.

## Publisher's Note

All claims expressed in this article are solely those of the authors and do not necessarily represent those of their affiliated organizations, or those of the publisher, the editors and the reviewers. Any product that may be evaluated in this article, or claim that may be made by its manufacturer, is not guaranteed or endorsed by the publisher.
